# CD146 is closely associated with the prognosis and molecular features of osteosarcoma: Guidance for personalized clinical treatment

**DOI:** 10.3389/fgene.2022.1025306

**Published:** 2022-10-21

**Authors:** Jingkun Wang, Zhonghan Wu, Meige Zheng, Shuisheng Yu, Xin Zhang, XinZhong Xu

**Affiliations:** Department of Orthopaedics, The Second Affiliated Hospital of Anhui Medical University, Hefei, China

**Keywords:** osteosarcoma, prognosis, microenvironment, immunotherapy, personalized treatment

## Abstract

**Background:** Osteosarcoma (OSA), a focus for orthopedic surgeons, always results in severe death due to metastasis. CD146 is severely expressed in several tumors, indicating its potential as a biomarker for OSA.

**Method:** Two OSA cohorts were enrolled in this study. A Therapeutically Applicable Research to Generate Effective Treatments-Osteosarcoma (TARGET-OS) cohort was used as a training cohort, and GSE21257 was used as the external validation cohort. The R package “limma” was used to discriminate the differentially expressed genes among CD146-high and CD146-low patients and was further annotated by the enriched signaling pathways. The R package MOVICS was used to evaluate immune infiltration and the response to chemotherapy and immunotherapy. All statistical analyses were performed by R version 4.0.2, and *p* < 0.05 was considered statistically significant.

**Result:** CD146 plays an important role in promoting the progression, invasion, and metastasis of several tumors. In the current study, we first revealed an integrative unfavorable prognosis in patients with tumors (*p* < 0.01, HR: 1.10, 95% CI: 1.07-1.14). CD146 is tightly correlated with m5C RNA methylation modification genes in OSA. Furthermore, we revealed that CD146 acts as an oncogene in OSA patients and is linked to poor prognosis in both the TARGET-OS cohort (*p* = 0.019, HR: 2.61, 95% CI: 1.171-5.834) and the GSE21257 cohort (*p* = 0.005, HR: 3.61, 95% CI: 1.474-8.855), with a total of 137 patients, regardless of whether they were adjusted for clinical pathological features. Highly-expressed CD146 impacts the signaling pathways of cytokine‒cytokine receptor interactions and is associated with the high infiltration of immunocytes. Moreover, patients with high CD146 expression were more likely to be sensitive to anti-PD-1 immunotherapy, while patients with low expression of CD146 were more likely to be sensitive to cisplatin and doxorubicin chemotherapy.

**Conclusion:** Overall, CD146 is an independent prognostic factor for OSA patients and can help doctors select clinical treatment strategies.

## Background

Osteosarcoma (OSA), which derives from primitive bone-forming mesenchymal cells, is the most common primary bone malignancy (Valery et al., 2015). The incidence rate of OSA changes by age, sex, and race. There are bimodal age peaks of OSA; one is at the age of 0–24 years old, and the other is over 65 years old (Mirabello et al., 2009a; Rojas et al., 2021). It was considered that the incidence of OSA in males was higher than that in females. Moreover, the incidence in females is higher than that in males in the group aged less than 15 years old (Nie and Peng, 2018). In addition, OSA is more likely to be observed in black people (6.8 per million persons per year) and Hispanics (6.5 per million persons per year) than in white people (4.6 per million persons per year) (Mirabello et al., 2009b; Ottaviani and Jaffe, 2009). Many factors can significantly affect the survival outcome of OSA, such as metastatic status, percentage of necrosis, tumor size, Enneking stage, local recurrence, and treatment therapeutic strategy (Pakos et al., 2009). Multidisciplinary approaches are applied for the treatment of patients with OSA—including surgery, radiotherapy, polychemotherapy, and immunomodulation—as 80%–90% of local OSA will develop metastasis (Ritter and Bielack, 2010). Although multidisciplinary treatment strategies have reduced mortality in OSA patients, the prognosis remains poor, and the 5-year and 10-year survival rates are only 56.31% and 22.33%, respectively (Yasin et al., 2020). Therefore, it is crucial to find robust biomarkers and prognostic factors to predict the progression, invasion, and metastasis of OSA.

Melanoma cell adhesion molecule (MCAM/CD146), which was reported in 1987 by Johnson and colleagues, is an integral membrane glycoprotein and belongs to the immunoglobin superfamily. It consists of a signal peptide, an extracellular fragment with five immunoglobin-like domains, a transmembrane region, and a short cytoplasmic tail (Lehmann et al., 1989; Johnson et al., 1993). At present, the pathological processes and physiological function of CD146 have been revealed, for instance, in tissue regeneration, inflammation, infections, signal transduction, cell migration, the cell cycle, mesenchymal stem cell differentiation, angiogenesis, and the immune response (Wang and Yan, 2013). As reported, CD146 is a cell surface receptor and can combine with many ligands involved in proliferation-related signaling pathways (Li et al., 2003). In addition, it can induce tumor angiogenesis (So et al., 2010). These biological characteristics promote tumor progression, invasion, and metastasis, especially in melanoma (Melnikova et al., 2009).

Currently, the focus on OSA has expanded from the tumor cell itself to the tumor microenvironment; the infiltrated immunocytes play an important role in tumor proliferation and migration or are resistant to chemo drugs (Corre et al., 2020). Tumor-associated macrophages account for approximately 50% of tumor volume of OSA (Huang et al., 2021), and M2 type can accelerate the process of tumor metastasis, which can be treated with all-trans retinoic acid to inhibit the polarization of M2 macrophage (Zhou et al., 2017). Yoshida et al. reported that anti-PD-1 therapy can inhibit the infiltration of Tregs with the murine LM8 cell osteosarcoma model, resulting in reduced tumor volume and prolonged survival (Yoshida et al., 2020). CD146 is highly expressed in advanced primary or metastatic melanoma cells but is rarely detected in normal cells, indicating its potential as a biomarker for predicting tumor initialization and prognosis. Beyond melanoma, increased expression of CD146 has been reported in more than ten types of tumors to date (Zabouo et al., 2009; Feng et al., 2012; Zeng et al., 2012; Tian et al., 2013; Wang and Yan, 2013; Li et al., 2014; Liang et al., 2017; Sechler et al., 2017). These studies have demonstrated that CD146 is a novel metastasis biomarker and prognostic factor and that it can also be used as a therapeutic target (Xie et al., 1997; Zabouo et al., 2009; Wu et al., 2012).

Similar results have been reported for OSA and CD146 (Schiano et al., 2012; Westrøm et al., 2016). In the current study, we confirmed the high expression of CD146 in most cancers, and the expression levels were significantly associated with the corresponding prognosis. Univariate and multivariate analyses were performed to reveal that CD146 acts as an independent prognostic factor for OSA patients. The critical pathways were identified by GO enrichment and HALLMARK analysis. Furthermore, we found that CD146 is a biomarker for anti-PD1 therapy. Our goal was to prove the prognostic value of CD146 in OSA and provide a novel tool for predicting immunotherapy efficacy.

## Materials and methods

### Data collection

Two OSA cohorts were enrolled in this study. The Therapeutically Applicable Research to Generate Effective Treatments (TARGET) osteosarcoma (TARGET-OS) cohort was derived from the UCSC Xena platform (http://xena.ucsc.edu/). It contains 84 samples of gene expression profiles and corresponding clinical information. The GENCODE27 annotation file was applied to annotate the gene symbols of mRNA. Another cohort, GSE21257, which contains 53 OSA patients, was downloaded from the Gene Expression Omnibus platform (GEO, http://www.ncbi.nlm.nih.gov/geo/). The TARGET-OS cohort was used as a training cohort, and GSE21257 was used as the external validation cohort. We conducted log2(TPM+1) processing on the expression data of CD146 to scale it. The median values of CD146 expression were set as threshold values to divide patients into low- and high-expression groups. In addition, we compared the expression of CD146 in normal and tumor tissues based on data from The Cancer Genome Atlas Program (TCGA) project (https://docs.gdc.cancer.gov/) and the GTEx project (https://www.gtexportal.org/home/). The evaluation of the CD146 prognostic value to overall survival (OS) time in pan-cancer was conducted on an online website (http://sangerbox.com/).

### Functional enrichment analysis

We used the R package “limma” to discriminate the differentially expressed genes (DEGs) among CD146-high and CD146-low patients (Ritchie et al., 2015). A fold-change > 0.4 and an adjusted *p* value <0.01 were set as the cut-off values to filter the DEGs. The packages “org.Hs.eg.db” and “msigdbr” were used to perform Gene Ontology (GO), Kyoto Encyclopedia of Genes and Genomes (KEGG), and HALLMARK enrichment analyses (Ashburner et al., 2000; Liberzon et al., 2015); clusterProfiler was applied to further elucidate potential gene functional interpretation and pathway enrichment (Yu et al., 2012).

### Immunocyte infiltration assessment

To evaluate the immunocyte infiltration status and the difference between the low- and high-expression groups, we collected 28 immunocyte signatures from prior research (Yoshihara et al., 2013). Subsequently, the runGSVA module in MOVICS was used to perform single sample gene set variation analysis (GSVA) to estimate the normalized enrichment score (NES) for the 28 signatures in each OSA patient (Lu et al., 2020).

### Efficacy prediction of chemotherapy and immunotherapy

We applied the comDrugen module in MOVICS to the clinical data of the TARGET-OS cohort to explore the chemotherapy efficacy difference between the low and high CD146-expression groups. Four chemotherapeutic drugs are shown in the violin plots. We also predicted the chemotherapeutic response to EBFs based on the largest publicly available pharmacogenomics database, the Genomics of Drug Sensitivity in Cancer (GDSC), with the use of the GSCA online website (Liu et al., 2018). To compare the likelihood of the CD146 groups with the immunotherapy subgroups, we performed subclass mapping analysis in the samples from patients who had received anti-PD-1 or anti-CTLA4 checkpoint therapy (Wang et al., 2016; Thanindratarn et al., 2019).

### Statistical analyses

All statistical analyses were performed by R version 4.0.2. Student’s t-test was applied to compare the two groups if the data were normally distributed for continuous data; otherwise, the Wilcoxon rank-sum test was used. For categorical data, the chi-square test and Fisher’s exact test were conducted. Kaplan‒Meier curves were generated to compare OS based on the log-rank test. A nomogram was constructed by the R package “regplot” with the results from Cox regression analysis. Calibration curves were plotted to assess the calibration ability of the nomogram, and decision curve analysis (DCA) was performed to show the clinical usefulness of the nomogram. The receiver operating characteristic (ROC) area under the curve (AUC) was calculated to assess the stability of prediction. To identify the independent risk factors, univariate and multivariate analyses were performed. We also established the Cox regression model to calculate the hazard ratio (HR) values and the 95% confidence interval (95% CI). The correlation was determined by the Pearson correlation test, with *p* < 0.05 being considered statistically significant.

## Results

### CD146 is highly expressed in pan-cancer tissues but expressed at low levels in normal tissues

**FIGURE 1:**
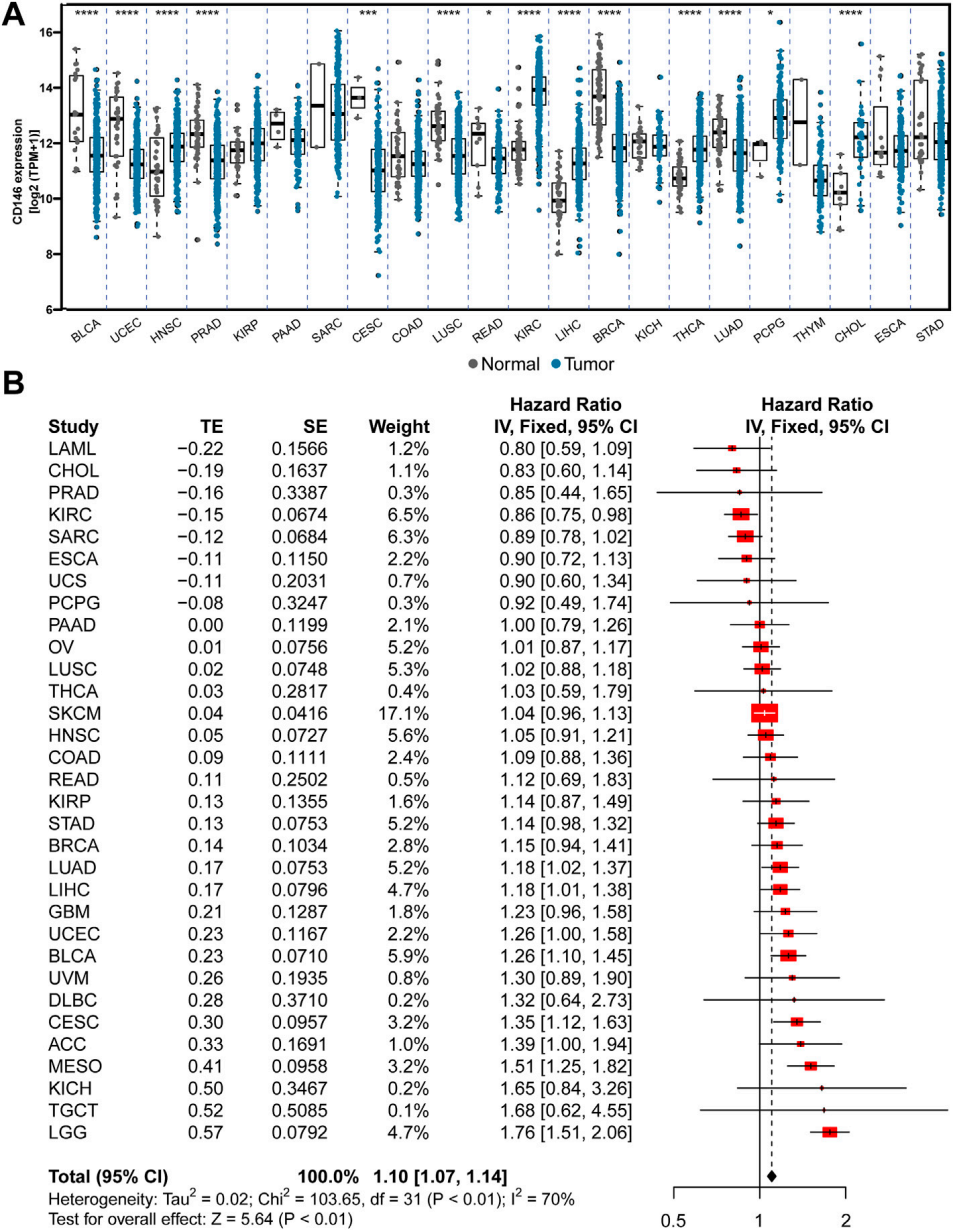
CD146 is increased in tumor tissue and predicts poor prognosis among pan-cancer. **(A)** The differential expression of CD146 in 22 types of tumor and adjacent normal tissues. **(B)** Meta-analysis revealed the integrative hazard risk of CD146 to pan-cancer.

Overexpression of CD146 has been reported in various cancers. However, the comparison of CD146 mRNA levels in different tumors is still unclear. Although all tumors have a certain CD146 mRNA level, the expression levels in sarcoma (SARC) and kidney renal clear cell sarcoma (KIRC) are higher than those in other tumors. The results also demonstrated that CD146 is highly expressed in tumor tissues relative to normal tissues in many tumors, including head and neck squamous cell carcinoma (HNSC), prostate adenocarcinoma (PRAD), kidney renal papillary cell carcinoma (KIRP), KIRC, liver hepatocellular carcinoma (LIHC), thyroid carcinoma (THCA), and pheochromocytoma and paraganglia (PCPG) (Figure 1A). Furthermore, we conducted a meta-analysis to evaluate the connection between CD146 expression and OS time across cancers. The results showed that CD146 acted as an oncogene and was linked to poor prognosis in most tumor types (*p* < 0.01, HR: 1.10, 95% CI: 1.07-1.14, Figure 1B). To validate the conclusion, we performed a log-rank test via Kaplan‒Meier curves, which was consistent with the meta-analysis (Supplementary Figure S1).

**FIGURE 2:**
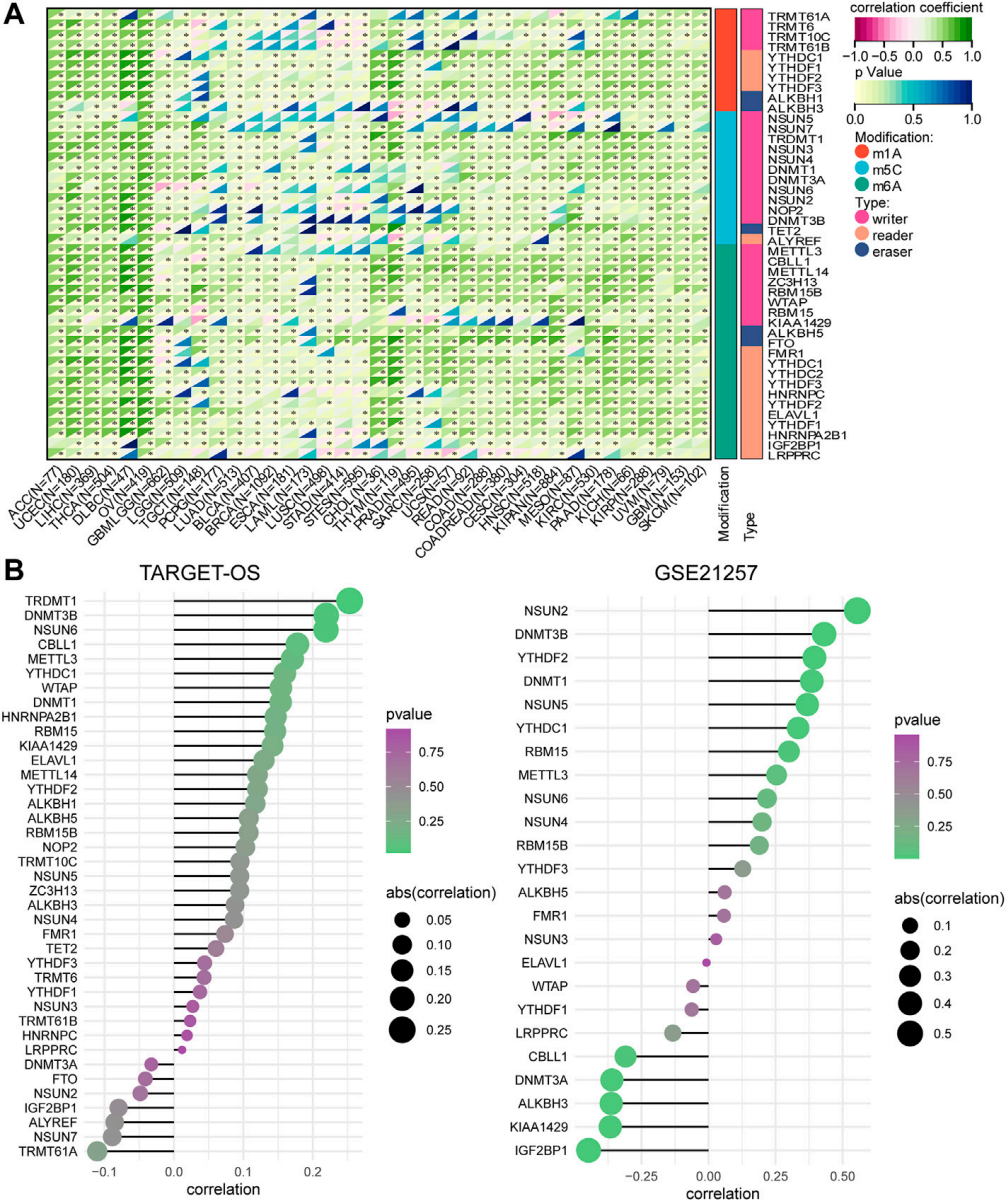
CD146 is regulated by RNA methylation. **(A)** Correlation of CD144 expression and RNA methylation regulators in m1A, m5C, and m6A among pan-cancer. **(B)** Correlation of CD144 expression and RNA methylation regulators among pan-cancer OSA datasets.

It is widely reported that posttranscriptional modification plays a key role in tumorigenesis, including the methylation modification methods of m1A, m5C, and m6A. Zhang et al. (2022) reported that METTL3 upregulates COPS5 expression by promoting COPS5 methylation in an m6A-related manner and results in the promotion of OSA progression. Li et al. (2022) also demonstrated the METTL14-mediated epitranscriptome modification of MN1 mRNA and the promotion of OSA tumorigenicity. Therefore, we assessed the correlation between RNA methylation modification genes and CD146 levels. We observed that the CD146 expression levels in most types of tumor positively correlated with RNA methylation modification based on the pan-cancer data from the TCGA project, especially for lymphoid neoplasms, diffuse large B-cell lymphoma, cholangiocarcinoma, ovarian serous cystadenocarcinoma, and thymoma (Figure 2A). For RNA methylation genes in OSA, we evaluated their correlation with CD146 in the TARGET-OS and GSE21257 cohorts and found similar results, especially for the m5C genes DNMT3B, NSUN2, NUSU5, NSUN6, and DNMT1 (Figure 2B).

### CD146 can act as a prognostic predictor and an independent prognostic factor for OSA

**FIGURE 3:**
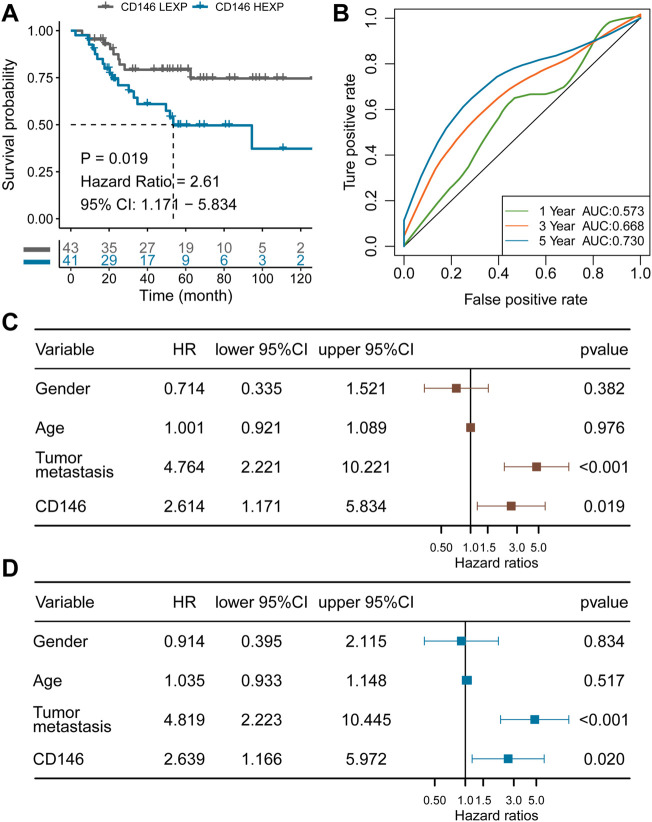
CD146 is an independent prognostic factor for OSA identified in the TARGET-OS cohort. **(A)** K-M plot showing the diverse clinical overall survival outcomes of the CD146 low and high groups; **(B)** ROC curve showing the prognostic value of the CD146 expression level; **(C)** results from univariate Cox regression analysis of the CD146 group and clinical features; **(D)** results from multivariate Cox regression analysis of the CD146 group and clinical features.

To explore the association between CD146 and OSA, we divided the TARGET-OS cohort into two subgroups: high CD146 expression (HEXP) and low CD146 expression (LEXP). The clinical feature distribution in HEXP and LEXP showed no difference, except for the first tumor event, and HEXP patients experienced more relapse (*p* = 0.014, Table 1). The Kaplan‒Meier curve for OS time is shown in Figure 3A. We found a statistically significant difference in OS time among patients in the HEXP and LEXP subgroups (*p* = 1.019, HR = 2.61, 95% CI: 1.71—5.834). As the 1-year, 3-year, and 5-year AUCs were 0.573, 0.668, and 0.730, respectively, we confirmed that CD146 is responsible for the poor prognosis in OSA (Figure 3B). Univariate analysis showed that the metastatic-diagnosis (*p* < 0.001, HR: 4.764, 95% CI: 2.221-10.221) and CD146 subgroups (*p* = 0.019, HR: 2.614, 95% CI: 1.171-5.834) were related to the OS time of OSA (Figure 3C). Multivariable analysis revealed that the same variables were related to poor prognosis, which proved that CD146 is an independent prognostic factor for OSA (*p* = 0.020, HR: 2.639, 95% CI: 1.166-5.972, Figure 3D). At present, the impact of age and sex on the prognosis of OSA is controversial (Pan et al., 2019; Ding et al., 2020). It is worth noting that we validated the impact of age and sex on prognosis to be small.

**TABLE 1:** Clinical features of the CD146 low and high groups in the TARGET-OS cohort.

Clinicopathological features		CD146 Low	CD146 high	*p* Value
Gender (%)	Female	15 (34.9%)	22 (53.7%)	0.130
	Male	28 (65.1%)	19 (46.3%)	
Age, years	—	15.55 ± 5.49	14.40 ± 4.00	0.273
Metastatic-Diag (%)	No	33 (76.7%)	30 (73.2%)	0.900
	Yes	10 (23.3%)	11 (26.8%)	
Race (%)	Asian	2 (4.7%)	4 (9.8%)	0.477
	Black or African American	4 (9.3%)	3 (7.3%)	
	Unknown	8 (18.6%)	12 (29.3%)	
	White	29 (67.4%)	22 (53.7%)	
First Event (%)	Censored	6 (14.0%)	5 (12.2%)	0.014*
	Death	1 (2.3%)	1 (2.4%)	
	No event	22 (51.2%)	9 (22.0%)	
	Relapse	13 (30.2%)	25 (61.0%)	
	SMN	0 (0.0%)	1 (2.4%)	
	Unknown	1 (2.3%)	0 (0.0%)	
Metastasis site (%)		32 (74.4%)	30 (73.2%)	0.796
	Bone and lung	2 (4.7%)	3 (7.3%)	
	Bone only	0 (0.0%)	1 (2.4%)	
	Lung only	9 (20.9%)	7 (17.1%)	
Primary tumor site (%)	Arm/hand	3 (7.0%)	3 (7.3%)	0.657
	Leg/foot	30 (69.8%)	32 (78.0%)	
	Leg/Foot	8 (18.6%)	6 (14.6%)	
	Pelvis	2 (4.7%)	0 (0.0%)	
Specific tumor site (%)	Arm NOS	0 (0.0%)	1 (2.4%)	0.097
	Femur	14 (32.6%)	24 (58.5%)	
	Fibula	5 (11.6%)	3 (7.3%)	
	Foot NOS	0 (0.0%)	1 (2.4%)	
	Humerus	2 (4.7%)	2 (4.9%)	
	Ilium	1 (2.3%)	0 (0.0%)	
	Leg NOS	4 (9.3%)	2 (4.9%)	
	Pelvis	0 (0.0%)	1 (2.4%)	
	Pelvis - ilium	0 (0.0%)	1 (2.4%)	
	Pelvis/Sacrum	1 (2.3%)	0 (0.0%)	
	Radius	1 (2.3%)	0 (0.0%)	
	Tibia	15 (34.9%)	6 (14.6%)	

**FIGURE 4:**
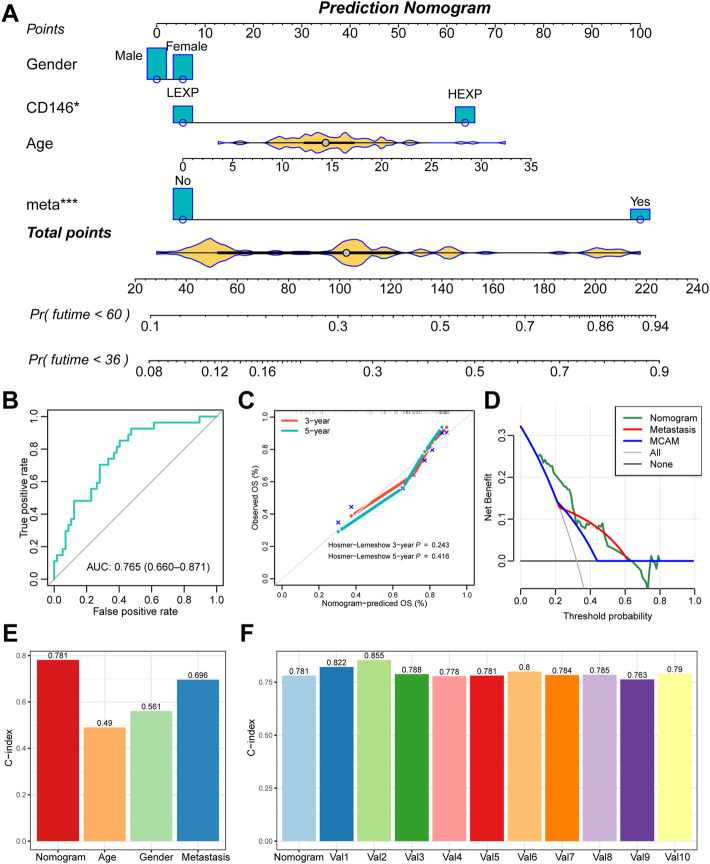
The nomogram further enhanced the prognostic value of CD146 in OSA. **(A)** Prognostic nomogram of 3-year and 5-year overall survival of OSA patients; **(B)** ROC curves for the nomogram of overall death events; **(C)** calibration plot to compare the nomogram prediction and the actual death events; **(D)** DCA curve for the nomogram, metastasis, and CD146 expression group; **(E)** C-index of the nomogram, age, gender, and metastasis status; **(F)** 10-times randomization C-index test to assess the stability of the prognostic nomogram.

Furthermore, we also constructed a prediction nomogram with the clinical features (Figure 4A). We revealed that the CD146 expression group acted as an assistor that enhanced the traditional pathological method. The total AUC value increased to 0.765 (95% CI: 0.660-0.871, Figure 4B), indicating the preferable prognostic value of the nomogram—also confirmed by the calibration curve. There was no significant difference between the nomogram-predicted survival probability and the actual survival probability (*p* = 0.243 for 3-year, *p* = 0.416 for 5-year, Figure 4C). We also observed that the nomogram performed with better prediction effectiveness than single metastasis or CD146 expression (Figure 4D). We calculated the prognostic C-index value of clinical features and observed that the nomogram contained the highest value of 0.781, as compared with age (C-index: 0.490), gender (C-index: 0.561), and metastasis status (C-index: 0.696) (Figure 4E). Moreover, we randomly selected a portion of patients (*n* = 60) to be re-assessed for the prognostic stability of nomogram ten times and observed that all the 10 C-indexes were higher than 0.75 (Figure 4F), indicating that the nomogram is accurate and stable.

### Mechanisms by which CD146 impacts OSA

**FIGURE 5:**
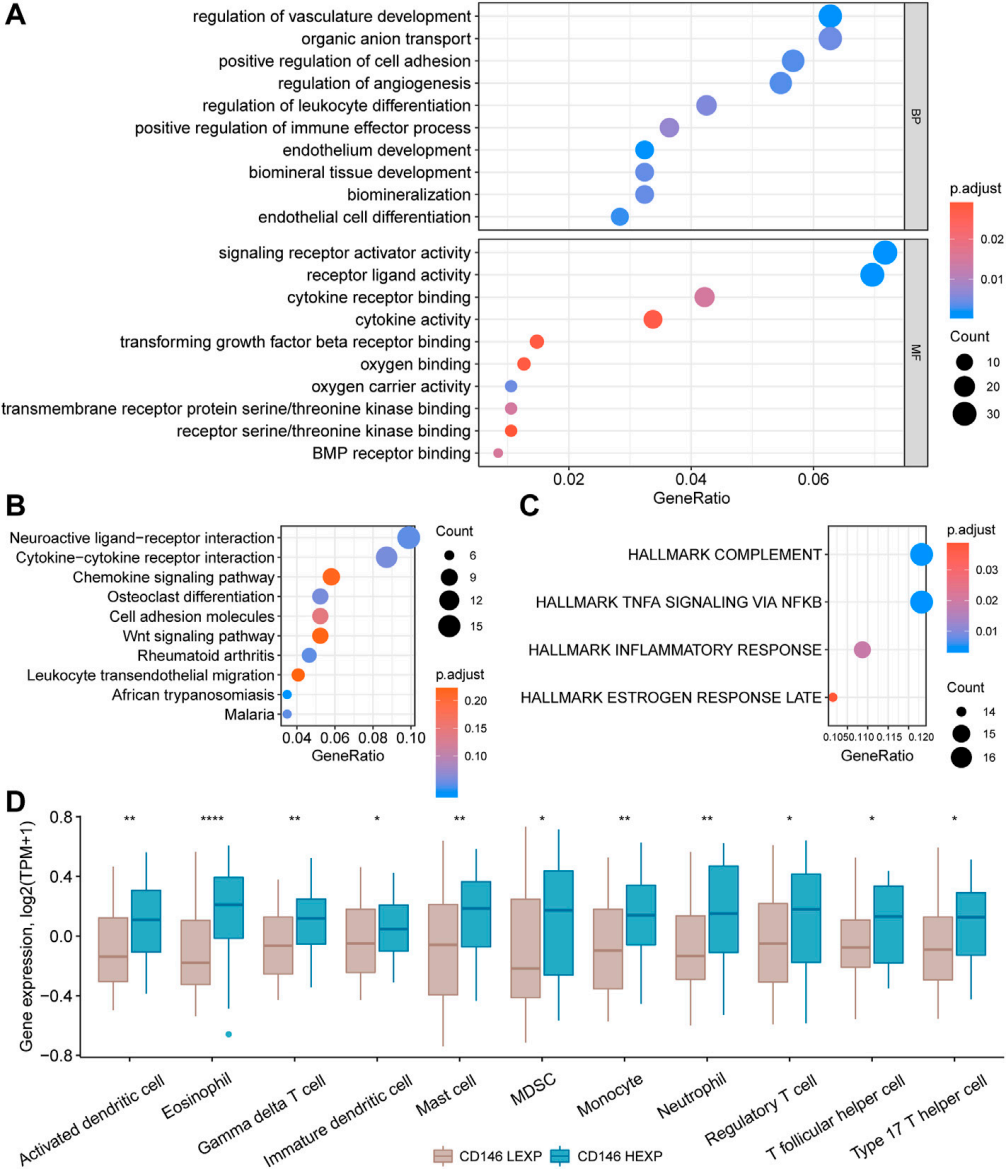
Enrichment of signaling pathways and differential infiltration of immunocytes in the CD146 high and low groups. Enrichment of 580 DEGs between the HEXP and LEXP subgroups by GO terms **(A)**, KEGG terms **(B),** and HALLMARK terms **(C)**. **(D)** Differential infiltration of 12 immunocytes between the CD146 high and low groups.

Enrichment analysis was carried out to investigate the potential mechanism by which CD146 impacts OSA. We first obtained 580 DEGs between the HEXP and LEXP subgroups along with the preset cut-off values mentioned above. The 580 DEGs were further enriched and annotated in different biological pathways. According to the biological process (BP) GO terms, pathways involving vasculature development and positive anion transport were the most relevant to CD146. In molecular function (MF) analysis, CD146 was obviously relevant to signaling receptor activator activity, receptor-ligand activity, cytokine receptor binding, and cytokine activity (Figure 5A). In KEGG analysis, numerous DEGs were enriched in the neuroactive ligand-receptor interaction and cytokine‒cytokine receptor interaction pathways (Figure 5B). For HALLMARK analysis, complement and TNFA signaling via NFKB had the most impact on CD146 (Figure 5C). The above enriched signaling pathways indicated that CD146 played an important role in the immune system in OSA. Therefore, we were concerned about the link between immunocytes and CD146 expression levels. As shown in Figure 5D, 11 types of immunocytes, including activated dendritic cells, eosinophils, gamma cells, immature dendritic cells, mast cells, monocytes, neutrophils, regulatory T cells, T follicular helper cells, type-17 T helper cells, and myeloid-derived suppressor cells were more highly infiltrative in the HEXP group.

### CD146 level used to choose the suitable precise clinical treatment for OSA patients

**FIGURE 6:**
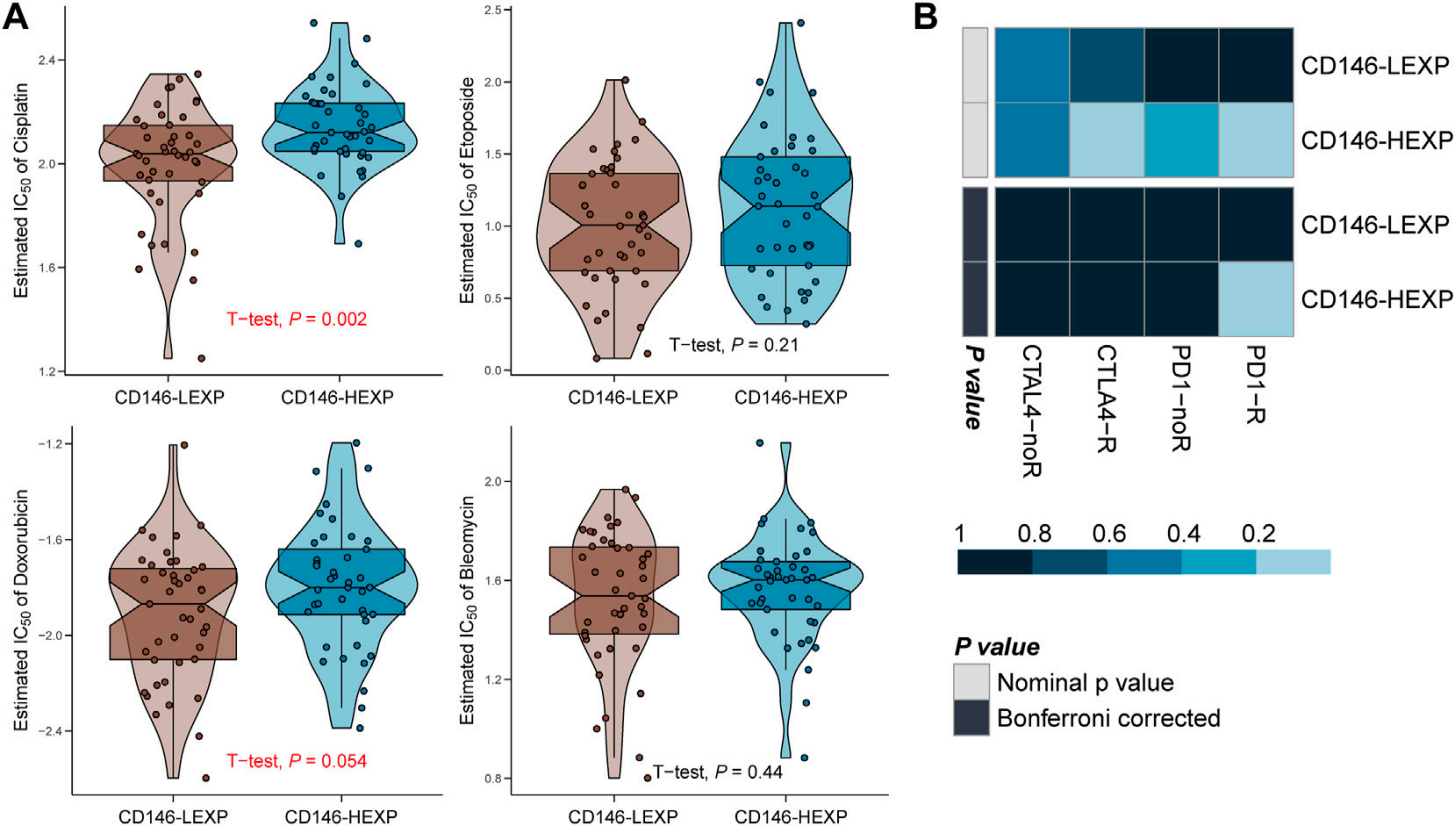
The CD146 level reflected the response to immunotherapy and chemotherapy. **(A)** The response of OSA patients to four common chemotherapy drugs, cisplatin, etoposide, doxorubicin, and bleomycin. **(B)** The response of OSA patients to anti-PD-1 and anti-CTAL4 immunotherapy.

Immunotherapy and chemotherapy have developed essential treatment strategies for excellent therapeutic effects on malignant tumors (Furue, 2003; Rodig et al., 2018). However, the current evidence shows that most checkpoint inhibitors have little effect on OSA and that chemotherapeutic drug resistance increases each year for multiple causes (Chatterjee and Bivona, 2019; Chen et al., 2021). Therefore, it is crucial to validate predictive biomarkers to optimize the choice of treatment strategy. In this study, we set the half maximal inhibitory concentration (IC_50_) as the observation index and compared the therapeutic effect of four commonly-used chemotherapy items in the LEXP and HEXP subgroups. We found a significant difference in the IC_50_ value of LEXP and HEXP to cisplatin (Figure 6A, *p* = 0.002) and that LEXP patients can respond to doxorubicin treatment (*p* = 0.054) but not to etoposide (*p* = 0.21) or bleomycin (*p* = 0.44) (Figure 6A). Furthermore, we also tried to select potential chemotherapy drugs from the GSCA online website and revealed that patients with high levels of CD146 were sensitive to chemotherapy with PLX4720, dabrfenib, SE590885, staurosporine, BRD-K99006945, vemurafenib, and several other proposed drugs (Table 2). To predict the immunotherapy response for each patient with OSA, we subsequently conducted SubMap analysis with an OSA cohort which included both patients receiving and not receiving anti-PD-1 or anti-CTLA4 therapy. There was no significant difference in CTAL-4 treatment response between the LEXP and HEXP subgroups; however, HEXP patients presented a better treatment response to anti-PD1 therapy than LEXP (Figure 6B, Bonferroni corrected *p* < 0.05).

**TABLE 2:** Prediction of potential drugs for CD146-high patients.

GDSC			
Symbol	Drug	Correlation	FDR value
CD146	PLX4720	−0.33871	9.43E-25
CD146	Dabrafenib	−0.29988	1.24E-17
CD146	SB590885	−0.25098	1.81E-11
CD146	(5Z)-7-Oxozeaenol	−0.24425	4.24E-12
CD146	MG-132	−0.24331	0.000315
CD146	selumetinib	−0.22606	3.49E-11
CD146	AZ628	−0.21711	0.000396
CD146	Bortezomib	−0.21643	0.000581
CD146	Trametinib	−0.20994	1.84E-09
CD146	PD-0325901	−0.20486	2.98E-08
CD146	RDEA119	−0.20124	4.27E-09
CD146	TGX221	−0.17799	0.0027
CD146	CHIR-99021	−0.17036	1.43E-06
CD146	CGP-082996	−0.16761	0.019578
CD146	CI-1040	−0.16183	2.99E-05
CD146	Dasatinib	−0.15004	0.015527
CD146	Docetaxel	−0.14687	6.23E-05
CD146	17-AAG	−0.1446	6.98E-05
CD146	Z-LLNle-CHO	−0.14257	0.028161
CD146	Bleomycin (50 uM)	−0.12835	0.000295
CD146	FH535	−0.12834	0.001947
CD146	Cisplatin	−0.12058	0.005306
CD146	piperlongumine	−0.11333	0.003932
CD146	Elesclomol	−0.10764	0.008985

CTRP			
CD146	staurosporine	−0.22964	0.017592
CD146	BRD-K99006945	−0.21083	0.002421
CD146	vemurafenib	−0.20254	3.17E-05
CD146	PLX-4720	−0.15145	0.005725
CD146	CIL41	−0.13905	0.033183
CD146	simvastatin	−0.13224	0.009889
CD146	lovastatin	−0.12506	0.004992
CD146	TGX-221	−0.12449	0.024848
CD146	AZD6482	−0.12091	0.013324
CD146	CAL-101	−0.11769	0.018051
CD146	XL765	−0.11361	0.024965
CD146	niclosamide	−0.10575	0.011619
CD146	TG-100-115	−0.10306	0.028828
CD146	fluvastatin	−0.10033	0.036151

### Validation of the prognostic value in the extra cohort

**FIGURE 7:**
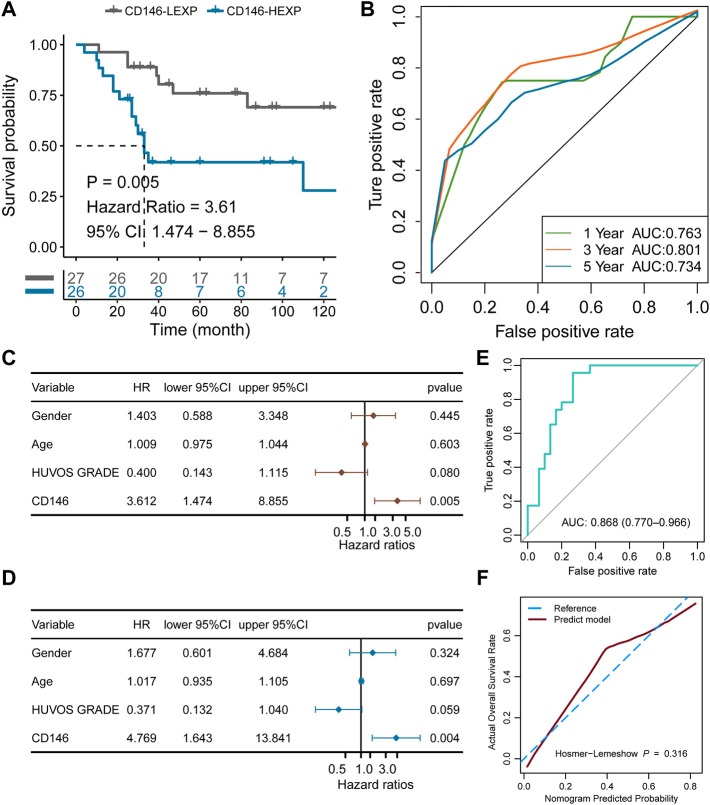
Validation of the independent prognostic value of CD146 in the external GSE21257 cohort. **(A)** K-M plot showing the diverse clinical overall survival outcomes of the CD146 low and high groups. **(B)** ROC curve showing the prognostic value of the CD146 expression level. **(C)** Results from univariate Cox regression analysis of the CD146 group and clinical features. **(D)** Results from multivariate Cox regression analysis of the CD146 group and clinical features. **(E)** Validation of the nomogram by ROC curve in GSE21257 cohort. **(F)** Validation of the nomogram by calibration curve in GSE21257 cohort.

To validate the value of prognostic prediction, the GSE21257 cohort containing 53 OSA patients was enrolled. We set the median value of CD146 expression as a cut-off value and divided the 53 OSA patients into HEXP (*n* = 26) and LEXP (*n* = 27) subgroups. The clinicopathological features among the two subgroups showed no difference, except a little among the metastasis status of tumor patients with high CD146 expression who met more tumor metastasis whether at or after diagnosis (*p* = 0.042, Table 3). Consistent with the results of the training cohort, the LEXP group showed higher OS, and the HEXP group had a 3.61-fold HR compared to the LEXP group (Figure 7A, 95% CI: 1.474-8.855, *p* = 0.005). The 1-year, 2-year, and 5-year AUCs were 0.763, 0.801, and 0.734, respectively, indicating reliability (Figure 7B). After removing the interference of confounding factors, we confirmed that CD146 was an independent prognostic factor (Figures 7C,D). For the prognostic nomogram generated in the TARGET-OS cohort, we calculated the point for each patient in the GSE21257 cohort; the ROC curve showed a high AUC value at 0.868 (95% CI: 0.770-0.966, Figure 7E). There was no significant difference between the nomogram-predicted survival probability and the actual survival probability (*p* = 0.316, Figure 7F).

**TABLE 3:** Clinical features of CD146 low and high groups in GSE21257 cohort.

Clinicopathological features		CD146 Low	CD146 high	*p* Value
Gender (%)	Female	11 (40.7%)	8 (30.8%)	0.638
	Male	16 (59.3%)	18 (69.2%)	
Age, years	—	18.43 ± 10.63	19.01 ± 13.85	0.866
HUVOS grade (%)	Grade 1	6 (22.2%)	7 (26.9%)	0.914
	Grade 2	9 (33.3%)	7 (26.9%)	
	Grade 3	7 (25.9%)	6 (23.1%)	
	Grade 4	3 (11.1%)	2 (7.7%)	
	Unknown	2 (7.4%)	4 (15.4%)	
Metastasis (%)	After diagnosis	7 (25.9%)	13 (50.0%)	0.042^a^
	At diagnosis	6 (22.2%)	8 (30.8%)	
	No	14 (51.9%)	5 (19.2%)	
Location (%)	Diaphysis of left femur	0 (0.0%)	1 (3.8%)	0.816
	Distal femur	0 (0.0%)	1 (3.8%)	
	Femur	3 (11.1%)	2 (7.7%)	
	Humerus	2 (7.4%)	2 (7.7%)	
	Left distal femur	6 (22.2%)	5 (19.2%)	
	Left femur	0 (0.0%)	1 (3.8%)	
	Left proximal femur	1 (3.7%)	0 (0.0%)	
	Left proximal fibula	0 (0.0%)	1 (3.8%)	
	Left proximal humerus	1 (3.7%)	1 (3.8%)	
	Left proximal tibia	5 (18.5%)	2 (7.7%)	
	Right distal femur	4 (14.8%)	2 (7.7%)	
	Right distal tibia	1 (3.7%)	0 (0.0%)	
	Right humerus	0 (0.0%)	1 (3.8%)	
	Right proximal femur	0 (0.0%)	1 (3.8%)	
	Right proximal fibula	1 (3.7%)	0 (0.0%)	
	Right proximal humerus	1 (3.7%)	0 (0.0%)	
	Right proximal tibia	1 (3.7%)	4 (15.4%)	
	Tibia	1 (3.7%)	1 (3.8%)	
	Unknown	0 (0.0%)	1 (3.8%)	
Histological (%)	Anaplastic	1 (3.7%)	1 (3.8%)	0.362
	Chondroblastic	2 (7.4%)	4 (15.4%)	
	Fibroblastic	4 (14.8%)	1 (3.8%)	
	Giant cell rich	0 (0.0%)	1 (3.8%)	
	Osteoblastic	15 (55.6%)	17 (65.4%)	
	Pleomorphic	1 (3.7%)	0 (0.0%)	
	Possibly chondromyxoid fibroma like	0 (0.0%)	1 (3.8%)	
	Sclerosing	1 (3.7%)	1 (3.8%)	
	Telangiectatic	3 (11.1%)	0 (0.0%)	

^a^ , *p* < 0.05.

## Discussion

Osteosarcoma (OSA) is one of the focuses of orthopedic surgeons. Although it is the most frequent bone cancer, the incidence rate among all tumor types is rare, with an annual incidence of 3–4 patients per million (Smeland et al., 2019). The metaphyses of long bones are vulnerable to OSA, including the distal femur, proximal tibia, and proximal humerus. Once OSA develops, patients typically present with swelling, pain, localized enlargement, and pathologic fracture. As a result of the low incidence rate and remarkable heterogeneity, the pathogenesis mechanism of OSA and prognostic factors remain unclear (Yang and Zhang, 2013). However, most patients are diagnosed at an advanced stage and die due to metastasis. Therefore, signatures with high accuracy and sensitivity for detecting metastasis and predicting survival are needed.

CD146 has been reported to be highly expressed in a variety of cancers in prior studies. CD146 plays an important role in promoting the progression, invasion, and metastasis of melanoma, gallbladder adenocarcinoma, and breast cancer. It has been confirmed as a predictor of poor survival in gastric cancer, lung adenocarcinoma, malignant pleural mesothelioma, and non-small cell lung cancer (Kristiansen et al., 2003; Sato et al., 2010; Liu et al., 2012; Jiang et al., 2016). Our study analyzed the expression of CD146 in 22 types of tumor and adjacent normal tissues and the correlation between CD146 mRNA levels and 32 types of tumor. We discovered that the mRNA level of CD146 was correlated with the corresponding prognosis. A high CD146 mRNA concentration indicated a poor survival time, especially in brain lower-grade glioma (LGG), testicular germ cell tumors (TGCT), kidney chromophobe (KICH), ACC, cervical squamous cell carcinoma and endocervical adenocarcinoma (CESC), lymphoid neoplasm diffuse large B-cell lymphoma (DLBC), and uveal melanoma (UVM)— consistent with previous studies. In addition, the expression levels of CD146 on OSA cell lines have been observed to be higher than those on normal osteoblast cells (OST) via confocal images (Schiano et al., 2012), implying its predictive value in OSA. We found that high CD146 expression was related to poor clinical outcomes in the training cohort: this result was validated in the GSE21257 cohort. Thus, we speculated that CD146 can act as a diagnostic biomarker to distinguish OSA from benign lesions. It is important to explore the mechanism of CD146 function in OSA. Lei et al. (2021) reported that CD146 promoted OSA growth by regulating angiogenesis and nourishment, endothelial cell proliferation, permeability, migration, vascular number, and diameter, which were similar to other tumors. However, the underlying mechanisms require further research. We enriched 580 DEGs in different pathways; as a result, vascular development, organic anion transport, cell adhesion, and ligand‒receptor interactions are likely involved in the development of OSA. Immunocyte infiltration has been reported in many cancers and is the signature of hot tumors, also named “immune-inflamed tumors”, which are more sensitive to immune checkpoint inhibitors (ICIs) than cold tumors (Meng et al., 2019). The most prominent characteristic of hot tumors is high T-cell infiltration (Liu and Sun, 2021). According to our study, OSA patients with high CD146 expression show abundant and diverse T-cell infiltration. Thus, we speculate that high CD146 expression is responsible for the sensitivity to immunotherapy. The hypothesis was confirmed through SubMap analysis. OSA patients with high CD146 expression were more vulnerable to anti-PD1 therapy. T-cell infiltration has also been reported as a prognostic factor. T follicular helper cells were proven to be capable of predicting pathological complete response after chemotherapy in breast cancer (Gu-Trantien et al., 2013). Nevertheless, we found that low CD146 was related to low T-cell infiltration but was also related to a high response to cisplatin therapy. More research is required in this field. Therefore, the high expression of CD146 groups represent subgroups with high immunocyte infiltration, low response to cisplatin but high response to anti-PD1 therapy, and poor prognosis; the low expression of CD146 groups represents subgroups with low immunocyte infiltration, high response to cisplatin but low response to anti-PD1 therapy, and good prognosis.

Previous studies have found many related factors affecting the prognosis of OSA patients. Age and sex were controversial in different studies. Some studies showed that the 5-year OS at 0–14 years old was lower than that in other age groups, while a meta-analysis showed no statistical correlation between age and the survival of OSAAs for sex. Some studies reported that the prognosis of male patients was worse than that of female patients, whereas some studies showed that sex had no correlation with prognosis. (Mirabello et al., 2009a; Joo et al., 2015; Ding et al., 2020). By performing robust statistical analysis, our study showed no correlation between age and the survival of OSA or sex. Multivariate analysis revealed that the CD146 mRNA level was an independent prognostic factor for OSA.

In summary, the role of CD146 in OSA was systematically analyzed. CD146 can act as a novel biomarker in predicting the prognosis of OSA and as an independent prognostic factor. We revealed the mechanisms by which CD146 promotes the growth, development, and metastasis of OSA. Contrary to cisplatin therapy, patients with high CD146 expression were more likely to be sensitive to anti-PD-1 therapy. The evidence above and prior research prove the potential value in individual survival prediction, in checkpoint therapy guidance, and as a novel therapeutic target (Stalin et al., 2017).

However, there are several limitations to our study. The samples we collected in the training and validation cohorts were insufficient. CD146 is a reliable prognosis-related factor but its role in the early diagnosis of OSA remains unclear. CD146 mRNA overexpression has been found in many cancers; therefore, the specificity for detection is poor. More research is needed in this field.

## Conclusion

We reveal that CD146 acts as an oncogene in the prognosis of several types of tumor and illustrate its function well in OSA. CD146 is an independent prognostic factor for OSA patients after adjusting for age, sex, and metastatic status at diagnosis. In addition, the expression of CD146 also indicates clinical treatment strategies. Patients with low expression of CD146 are more suitable for chemotherapy with cisplatin and doxorubicin, while patients with high expression of CD146 respond better to anti-PD-1 immunotherapy.

## Data Availability

The original contributions presented in the study are included in the article/Supplementary Material; further inquiries can be directed to the corresponding author.
