# Supplementary figures and images for: CD146 is closely associated with the prognosis and molecular features of osteosarcoma: Guidance for personalized clinical treatment
Source: Front Genet. 2022 Oct 21;13:1025306. doi: 10.3389/fgene.2022.1025306 (PMC9635853; doi:10.3389/fgene.2022.1025306)

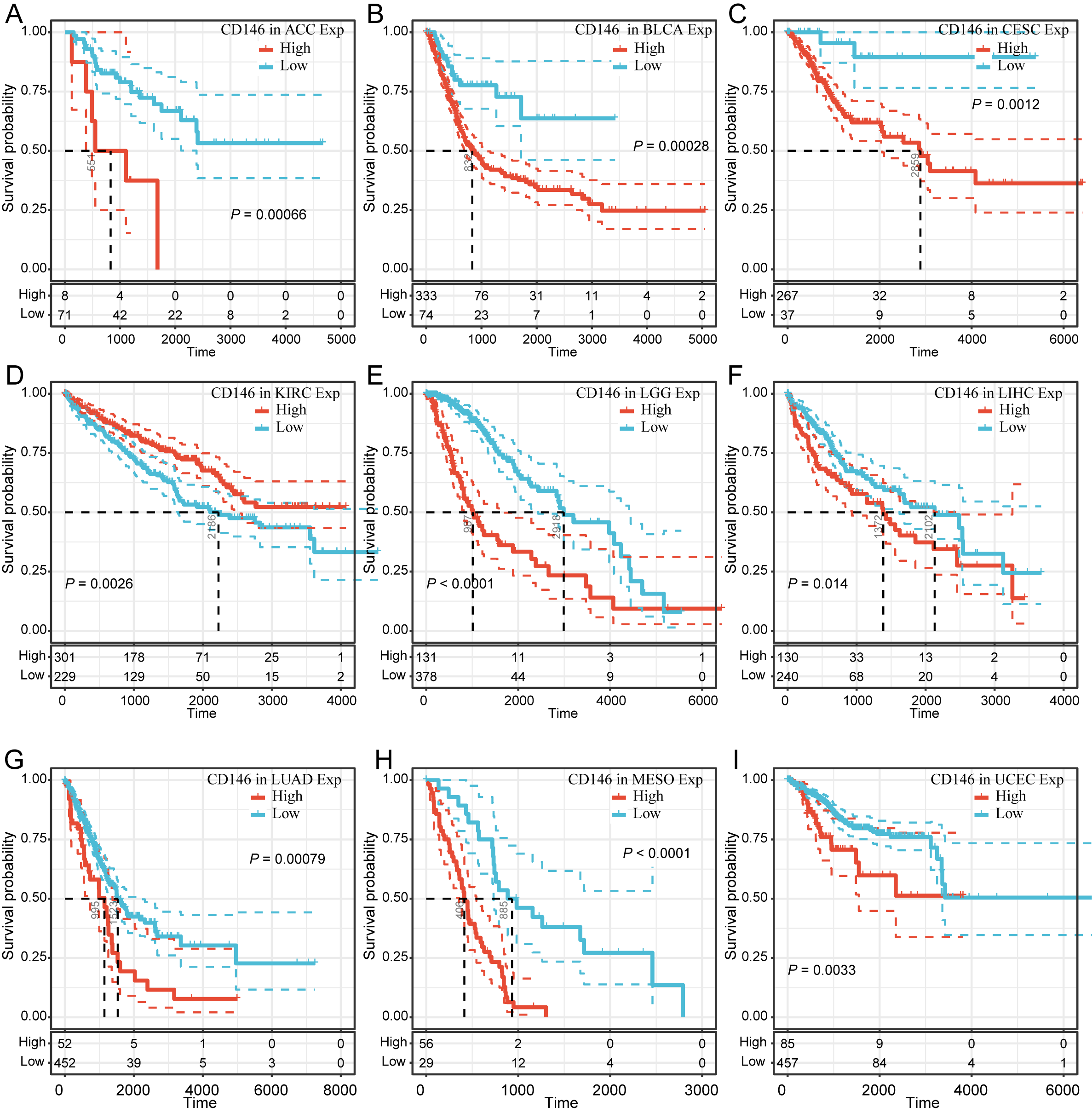

Supplement: Supplementary file 1 [file Image1.TIF]
